# Spectral phenotyping of embryonic development reveals integrative thermodynamic responses

**DOI:** 10.1186/s12859-021-04152-1

**Published:** 2021-05-06

**Authors:** Oliver Tills, John I. Spicer, Ziad Ibbini, Simon D. Rundle

**Affiliations:** Marine Biology and Ecology Research Centre, School of Biological and Marine Sciences, University of Plymouth, Drake Circus, Plymouth, PL4 8AA Devon UK

**Keywords:** Invertebrate development, Aquatic embryo, Energy proxy traits, High dimensional organismal phenotyping

## Abstract

**Background:**

Energy proxy traits (EPTs) are a novel approach to high dimensional organismal phenotyping that quantify the spectrum of energy levels within different temporal frequencies associated with mean pixel value fluctuations from video. They offer significant potential in addressing the phenotyping bottleneck in biology and are effective at identifying lethal endpoints and measuring specific functional traits, but the extent to which they might contribute additional understanding of the phenotype remains unknown. Consequently, here we test the biological significance of EPTs and their responses relative to fundamental thermodynamic principles. We achieve this using the entire embryonic development of *Radix balthica*, a freshwater pond snail, at different temperatures (20, 25 & 30 °C) and comparing responses against predictions from Arrhenius’ equation (Q_10_ = 2).

**Results:**

We find that EPTs are thermally sensitive and their spectra of frequency response enable effective high-dimensional treatment clustering throughout organismal development. Temperature-specific deviation in EPTs from thermodynamic predictions were evident and indicative of physiological mitigation, although they differed markedly in their responses from manual measures. The EPT spectrum was effective in capturing aspects of the phenotype predictive of biological outcomes, and suggest that EPTs themselves may reflect levels of energy turnover.

**Conclusions:**

Whole-organismal biology is incredibly complex, and this contributes to the challenge of developing universal phenotyping approaches. Here, we demonstrate the biological relevance of a new holistic approach to phenotyping that is not constrained by preconceived notions of biological importance. Furthermore, we find that EPTs are an effective approach to measuring even the most dynamic life history stages.

**Supplementary information:**

The online version contains supplementary material available at 10.1186/s12859-021-04152-1.

## Introduction

Technology-enabled high-dimensional-organismal-phenotyping (HDOP) provides efficient measurement of phenotypic traits traditionally used in biology, and can also extend the study of the phenotype via proxy traits—measures with no direct traditional equivalent [1–5]. A new approach to measuring the phenotype is the use of energy proxy traits (EPTs), the amount of energy within different temporal frequencies in the pixel value fluctuations from video of live biological material. Analysis of fluctuations in video pixel values has previously been used to measure cardiac activity in humans [6], model species [7, 8] and aquatic invertebrate embryos [5]. While EPTs also have pixel value fluctuations as their basis, they are a holistic approach to measuring complex biological responses not limited by preconceived notions of the phenotype and the traits used to quantify it. Such holistic approaches to quantifying the phenotype while holding significant potential in advancing the concept of phenomics, are relatively scarce.

Unlike many automated methods for measuring the phenotype, EPTs do not require targeting of any particular aspect of an organisms’ morphology, physiology or behaviour (e.g. net movement). Rather, they are temporal frequency spectra, capturing fluctuations in mean pixel values, a simple image summary statistic, arising from any biological source visible in video. EPTs have proven an effective approach to the study of aquatic embryos, including incorporation of the ontogeny of heart function, muscle movement, rotation and net movement of the embryo within EPT datasets [5]. The application of spectral frequency analysis has been demonstrated to be an effective method for interrogating complex biological responses in applications including movement ecology [9] and movement analysis of numerous species of aquatic embryos [10, 11].

Time series analysis of these spectral data, from successive developmental time points, has proven effective in quantifying the holistic response of aquatic embryos during the process of biological development within different temporal frequency bins. Targeted analyses within EPT datasets have proven effective at extracting and quantifying signals from specific ‘traditional’ components of an embryo’s developmental physiology, including lethal end points, and heart rate in aquatic invertebrates with very different heart architectures (i.e. molluscs and amphipods [5]). Indeed, aquatic embryos and larvae are emerging as scalable models for the use of HDOP, with technologies and methodologies increasingly becoming available [2, 5, 12]. Early use of EPTs in aquatic embryos has found that they are sensitive to different environmental conditions [5], but their mechanistic underpinning and biological relevance remains unclear.

Here, we investigate the mechanistic underpinning of EPTs and compare them with traditional measures of whole-organismal biology. We do this by comparing measures of EPTs within a thermodynamic context. Temperature drives the rate of biochemical reactions and biological processes in ectotherms and so is one of the most important environmental factors in the genome-to-phenome relationship [13, 14]. The temperature sensitivity of rates and reactions can be predicted using Arrhenius’s Eq. [15] and expressed using a relatively simple temperature coefficient, Q_10_. Without any physiological alteration (i.e. physiological adapation, sensu Prosser (1958) [16] but without any inference of positive fitness benefit) organismal responses should exhibit a temperature coefficient of two (Q_10_ = 2), i.e. a doubling of rate for a 10 °C increase in temperature. We used EPTs to quantify the response of embryos of a pond snail, *Radix balthica*, to different thermal environments, specifically to: i) assess concordance of EPTs with thermodynamic predictions using calculated Q_10_ s; and ii) compare EPTs with traditional biological measures. We also investigated whether: iii) the combined responses of EPTs were thermally specific or simply a function of the sensitivity of individual traits; and iv) levels of EPT during embryonic development were predictive of developmental outcomes.

## Results

### Energy proxy traits show unique deviations from thermodynamic predictions

**Fig. 1:**
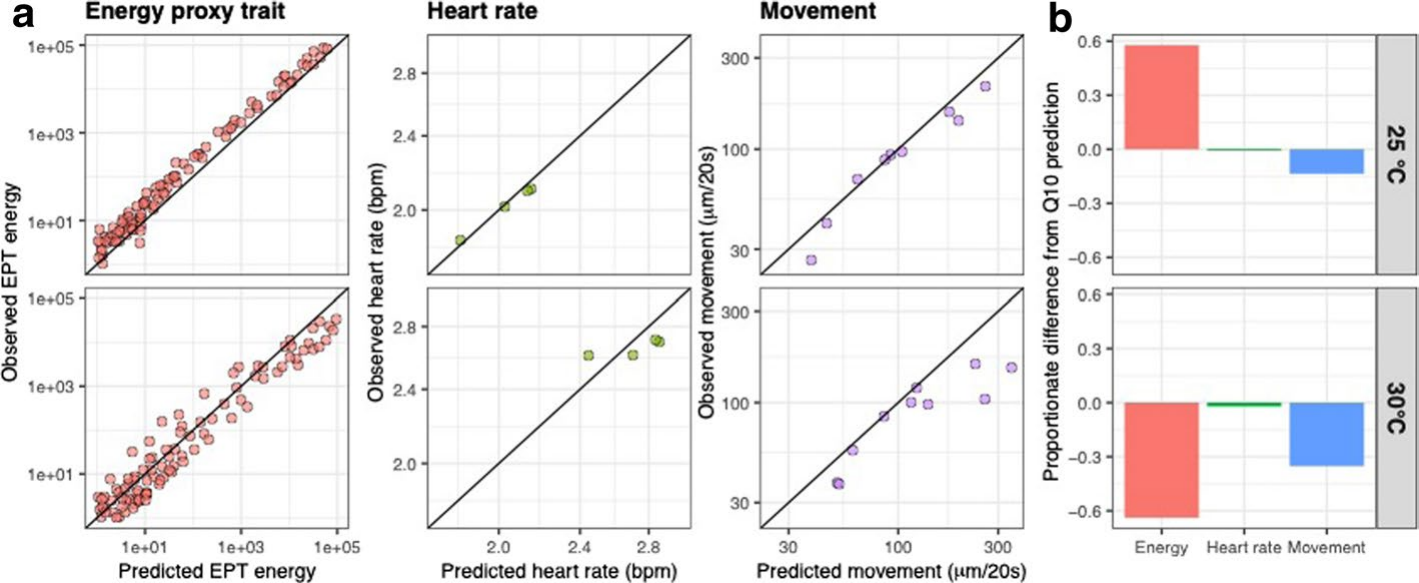
**a** Observed developmental-stage specific phenome responses for overall levels of different frequencies of EPT, heart rate and overall movement compared with the predicted response (Q_10_ = 2, applied to the observed response at 20 °C). **b** Proportionate deviation in the response of the phenome-level traits in embryos at 25 °C and 30 °C from Q_10_ predictions

Thermodynamic predictions of responses at 25 and 30 °C were calculated on the basis of Q_10_ = 2 applied to the responses observed at 20 °C. Concordance between observed levels of EPT and thermodynamic predictions were evident at both 25 and 30 °C and across the full range of frequencies measures (Fig. 1a), but with some exceptions. There was a significant difference between temperatures in the number of EPTs exhibiting either positive or negative deviation from Q_10_ = 2 predictions (χ_1_ = 69.06, p ≤ 0.001, Additional file 1, 2). At 25 °C, 94% of EPTs were greater than predicted, whereas at 30 °C, 68% of EPTs were lower than predicted. Furthermore, the proportionate difference in EPT predictions was significantly different between 25 °C and 30 °C (F_1,258_ = 114.7, P ≤ 0.0001) with an approximate 60% increase at 25 °C compared to a 60% decrease at 30 °C (Fig. 1b, Additional file 3). Levels of variation in EPTs was greater at 30 °C than at 25 °C, indicated by an increased scatter around the line of equilibrium indicative of Q_10_ = 2.

Responses of both net movement of the embryo within the egg and heart rate were more closely aligned to Q_10_ = 2 predictions, compared to EPTs (Fig. 1b). There was no significant difference between temperatures in the number of traditional trait measurements above or below predicted values, or in the proportionate difference of responses between temperatures (heart rate: proportional difference—F_1,6_ = 0.157, P = 0.706; count—χ_1_ = 0, p = 1. movement: proportional difference—F_1,18_ = 2.32, P = 0.145; count—χ_1_ = 1.57, p = 0.21).

**Fig. 2:**
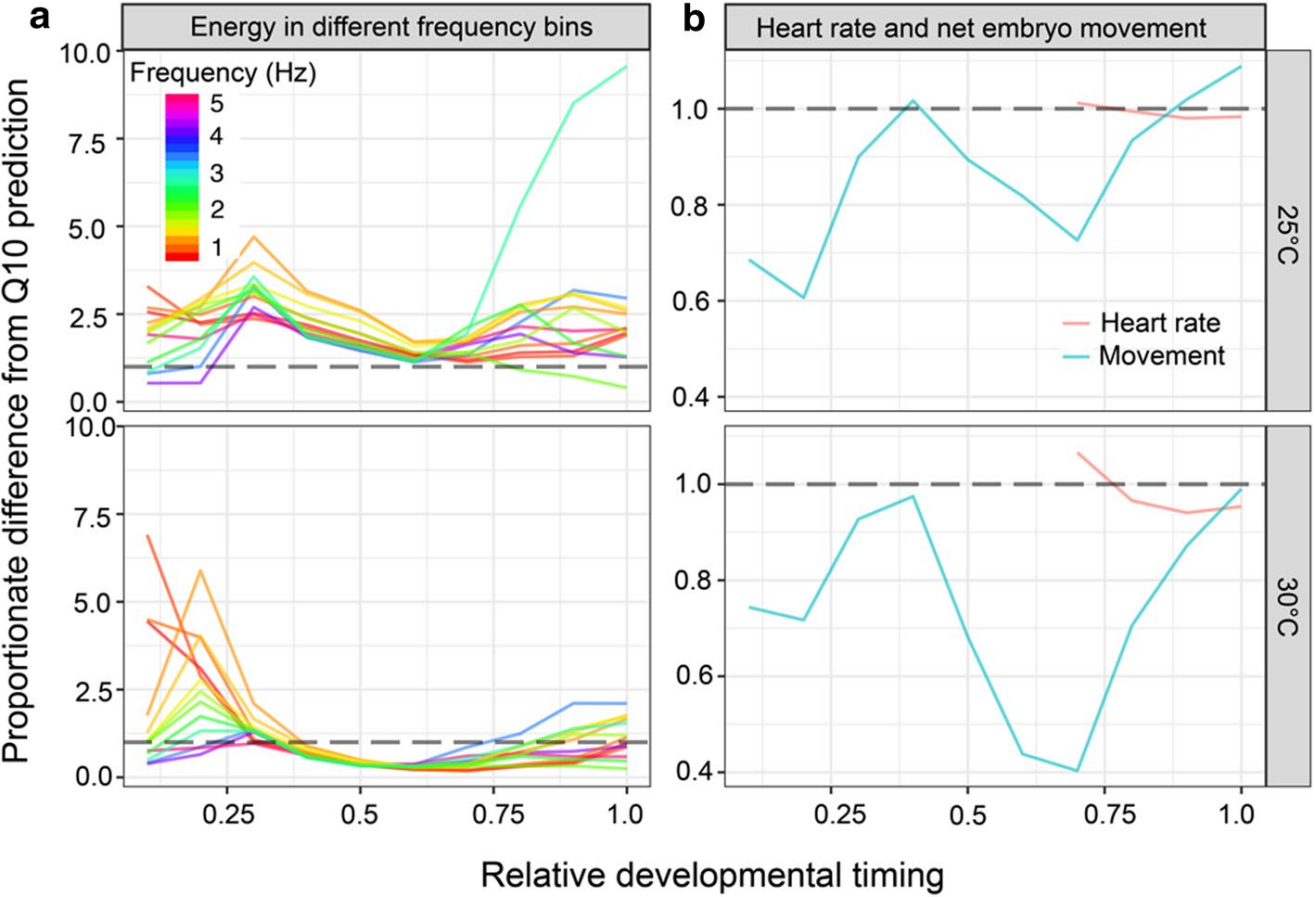
**a** Developmental stage-specific deviation from thermodynamic predictions (Q_10_ = 2 in energy proxy traits within 12 frequency bins across ten developmental periods, based on thermodynamic predictions. Alignment to Q_10_ = 2 prediction is indicated by the horizontal dashed lines within each panel. **b** Developmental period-specific difference in overall movement and heart rate from thermodynamic predictions. Alignment to Q_10_ = 2 prediction is indicated by the horizontal dashed line in each panel

Assessment of the magnitude of deviation from thermodynamic predictions during the course of embryonic development was used to assess the relative thermal sensitivity of EPTs within different developmental periods (Fig. 2). Deviation of EPTs from thermodynamic predictions was highly temperature and developmental-period specific, with a significant interaction between the two across frequencies (Fig. 2; temperature—F_1, 73_ = 192.06, P ≤ 0.0001, relative developmental timing – F_9, 9332_ = 94, P < 0.0001, temperature*relative developmental timing – F_9, 9332_ = 36.23, P ≤ 0.0001, Additional file 4). At 30 °C, there was a marked reduction in all frequencies of EPT when approximately 30% of embryonic development had elapsed. During this period the embryo undergoes a major locomotory transition, from ciliary-driven rotation to the use of the foot for settlement onto the wall of the egg before crawling [17]. Levels of EPT returned close to previous values in most frequencies for the final 20% of embryonic development, however EPTs at 3 Hz were significantly greater than predicted and this corresponds to the heart rate of the embryo during the later stages of development. The same increase of EPT associated with the development of cardiac function was evident at 25 °C, but at a lower frequency of 2 Hz.

Similar to EPTs, deviation in gross levels of embryonic movement within the egg capsule from thermodynamic predictions were significantly affected by temperature, developmental period and their interaction (temperature F_1,73_ = 38.69, P ≤ 0.0001; developmental period F_9, 620_ = 21.55, P ≤ 0.0001, temperature*developmental period F_9,620_ = 6.62, P ≤ 0.0001). In contrast to EPTs and gross movement, there was no effect of temperature on the deviation of cardiac function from thermodynamic predictions, but there was an effect of developmental period (temperature – F_1, 72_ = 2.3, P = 0.1334, developmental period – F_3, 188_ = 13.65, P ≤ 0.0001).

### Temperature-specific combinatorial energy proxy traits evident throughout development

**Fig. 3:**
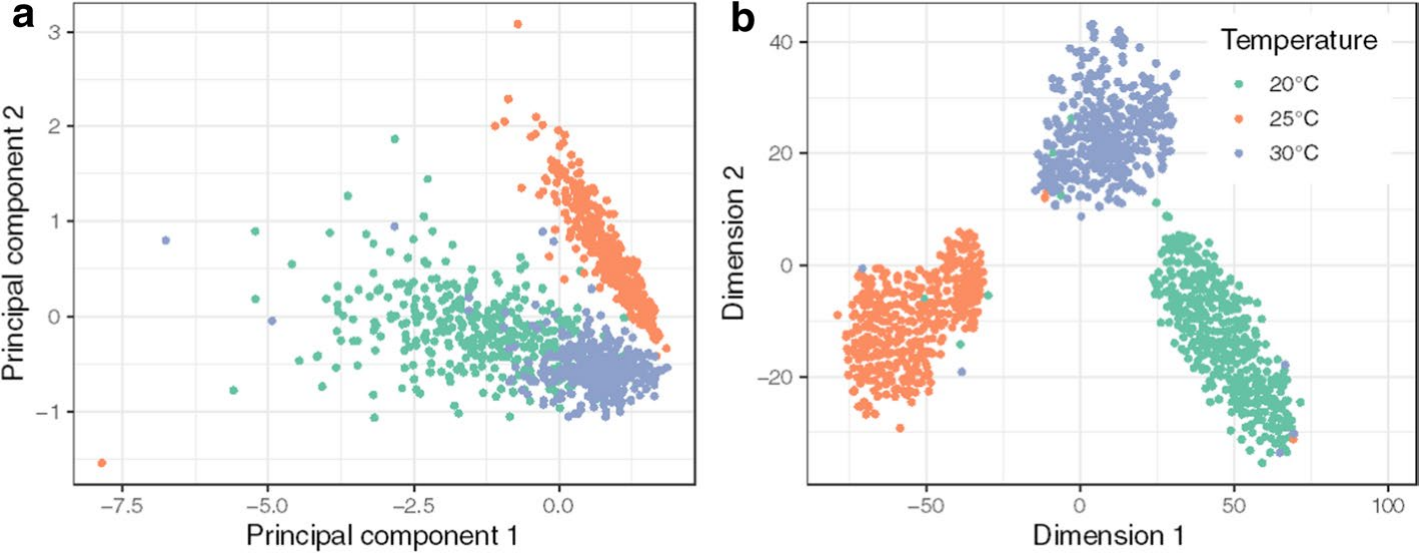
**a** Principal component analysis (PCA) of the levels of energy within 150 frequency bins at each hourly time point for the duration of embryonic development of *R. balthica*. **b** T-distributed stochastic neighbour embedding clustering of each hourly time point from the first 50 principal components from a PCA on the basis of hourly frequency-specific energy levels (PCA-tSNE)

To determine the multivariate response of EPTs to different temperatures, principal component analysis (PCA) was applied to the mean energy proxy trait values for each frequency for hourly developmental timepoints (Fig. 3a). A multivariate analysis of variance was applied to the first twenty principal components (PCs). PCA was effective in capturing 96% of the variance in the EPTs and was significantly different between temperatures (Pillai’s Trace = 0.832, F_1,1074_ = 265.12, P ≤ 0.001). Subsequent *post-hoc* univariate analysis identified PCs 1–5 and PC19 as being significantly different between different temperatures (Additional file 5). While there were significant differences in several PCs between temperatures, some outlying timepoints are visible in their projection along PCs 1 and 2 and this appears to result from asynchronous hatching meaning that later timepoints are more greatly impacted by inter-individual variation in EPT profiles. PCA is a linear dimensionality reduction method, and so, in order to better understand the thermal sensitivity of EPTs, PCA was used in combination with t-SNE (PCA-tSNE), a non-linear method for visualisation of high-dimensional datasets. PCA-tSNE revealed even greater clustering between temperatures and indicates that the higher dimensions captured in the PCA (beyond PCs 1 and 2) contribute to capturing biologically relevant, treatment-specific responses (Fig. 3b, Additional file 6).

### Levels of energy proxy trait are predicted by growth rate

**Fig. 4:**
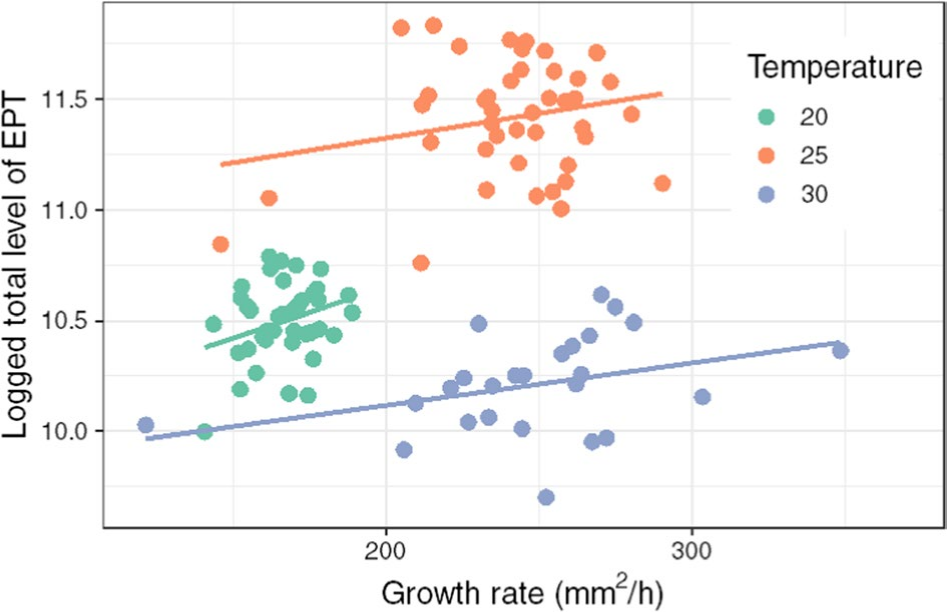
Growth rates of *R. balthica* cultured in different thermal regimes (°C) and their corresponding overall levels of energy derived from developmental EPT data

To investigate the broader significance of EPTs in terms of their relation to developmental outcomes, relationships between total EPT levels and growth rates were explored for individual embryos at different temperatures using ANCOVA (Fig. 4). Levels of EPT were significantly different between temperatures (F_2, 105_ = 278.8, P ≤ 0.0001; 20 vs 25 °C—P ≤ 0.0001, 20 vs 30 °C P ≤ 0.0001) and as predicted, so too were growth rates (F_2, 105_ = 151.2, P ≤ 0.0001; Additional file 7). However, there was also a significant positive relationship between the overall levels of EPT measured throughout an embryo’s development and its growth rate (F_1, 104_ = 6.09, P = 0.0153) in all three temperatures studied.

## Discussion

Energy proxy traits are a holistic approach to measuring the phenotype during arguably the most dynamic life stage – embryonic development. Here, we show that EPTs are thermally sensitive throughout development, exhibit deviations from biochemical prediction that are indicative of physiological alteration, and that overall levels of EPT are predicted by a key developmental outcome, growth rate.

The divergence of observed values from those predicted on a purely thermodynamic basis was significantly different between EPTs and manual measures of gross movement and heart rate. Movement and heart rate are both dominant components of the observable physiology of the embryonic stage of the aquatic gastropod *Radix balthica* and were lower at 25 °C than predicted. In contrast, the deviation in the response of EPTs was higher, i.e. levels of EPT were greater than purely thermodynamic predictions. Differences in the thermal response of different phenotypes has been recorded previously [14]. Here, the differences in thermal response between EPTs and the manual measures of both heart rate and movement reinforces the importance that phenotypic expression can have in determining the outcomes of experiments. Energy proxy traits are not targeted towards a particular aspect of the developmental phenotype, but instead indiscriminately capture the physiological responses that are detectable within pixel fluctuations. We suggest that the additional information content of EPTs means that they could have significant potential in measuring and integrating phenotypic responses in a way that is not otherwise be possible.

Deviations from Arrhenius predictions are indicative of responses not being solely driven by thermodynamic effects, but instead indicate some degree of physiological alteration, possibly adaptation, occurring [15]. For EPTs, such physiological alteration, is underpinned by changes in physiology contributing to frequency-specific levels of energy. We routinely undertake a reductionist approach to biology, whereby our level-of-observation determines where we interrogate and interpret biological responses [3, 18, 19]. The non-prejudiced and integrative nature of EPTs contrasts sharply with many of the methods commonly used to study the phenotype. This could go some way to explain the contrasting thermal responses observed here. While integrative analyses of molecular omics are now commonplace, phenotype measurements are typically only used for interpretable biological phenomena for which mechanisms are understood, at least to some level. However, we are now at the exciting stage of being able to broaden our understanding of the phenome beyond traditional phenotypes and the use of EPTs could be a major contribution to this.

The diversity and dynamics of form and function during embryonic development has always presented a challenge to biologists [20]. The same is true in building effective approaches for HDOP [5]. Here, we show that the developmental trajectories of EPTs altered significantly during ontogeny and these changes were temperature- and frequency-specific. Developmental trajectories in EPTs were effective in capturing the biology of developmental stages with very different phenotypes, but importantly they could also continue this measurement during the transition between these stages. Developmental transitions in *R. balthica* include a change from ciliary propelled spinning and gliding to a muscular crawling behaviour (Additional file 8), and the ontogeny of cardiac function; traditional phenotypic measures are largely ineffective or not transferrable between these periods. Universality in the types of measurement that can be applied to different systems, including species, developmental stages, and experimental designs, could enable more readily accessible and transferrable technologies across biology. Previous research has demonstrated the applicability of EPTs to different species and to different types of experimental design, including studying the effects sub-lethal, lethal and multi-drivers [5].

Dimensionality reduction enables the extraction of structure from complex high-dimensional datasets and is a well-established method in biology for interrogating complex datasets, and this includes the serial combination of different dimensionality reduction methods [21–23]. Dimensionality reduction of EPTs generated combinatorial signals that were highly effective in clustering embryos throughout their development on the basis of treatment temperature. For 20, 25 and 30 °C the first five principal components from a principal component analysis were significantly different. The first two principal components accounted for 63% of the variance in the EPTs and therefore to understand the contribution of the additional variance we applied t-SNE (PCA-tSNE), a non-linear clustering method, to the eigenvectors from the first 50 principal components produced by the PCA. PCA-tSNE enabled the integration of a greater portion of the variance present in the EPT data (99%) and this revealed tight clustering of individual hourly time points by temperature treatment.

The growth rate of embryos was positively related to the overall level of EPT measured throughout embryonic development at different culture temperatures. Variation in growth rate linked to levels of EPT is important as it is suggestive of changes to whole-organism physiology with potential fitness implications – either via accelerated development, or a larger size at hatch. A positive relationship between EPT and growth rate would appear to support the hypothesis that EPTs could be a good proxy for metabolic rate. Intra- and inter- individual variation in metabolic rate varies significantly during embryonic development. Therefore, the inter-individual variation in EPTs, if representative of metabolic rate, would not be unusual [24]. If EPTs are a good proxy for metabolic rate this will enable the extension of whole-organismal metabolic rate, to energy turnover within different temporal frequencies. Furthermore, the measurement of EPTs is a simpler and lower cost method than respirometry and can be achieved with less manipulation of the treatment environment [5].

At 25 °C, there were significantly greater levels of EPT than at either 20 °C or 30 °C. At 30 °C EPTs were significantly lower than predicted and were almost lower than in 20 °C. This was confirmed by manual observation of the video which showed that at 25 °C, embryos during the last two thirds of embryonic development had generally greater levels of muscular movement than observed at 30 °C. Conversely, while heart rate was significantly higher at 30 °C than at 25 °C, other physiological aspects appeared subdued. The application of EPTs as a method to quantify the entire period of embryonic development enabled the acquisition and analysis of a continuous physiological time series. A lack of universality in traditional phenotypic measures represent a key limitation to the more widespread adoption of HDOP across biology. Further research is now needed to apply EPTs to addressing key physiological and evolutionary questions in early life stages to extend our understanding of how they can contribute to our understanding of phenotypic responses more broadly in biology.

## Materials and methods

### Experimental approach

The development of 144 embryos of a freshwater gastropod, *Radix balthica,* were recorded using EmbryoPhenomics, a platform for HDOP [5], at temperatures of 20 n=42, 25 n=44 and 30 n=32 °C [25]. Individual embryos were imaged (200 × magnification, 750 × 750 pixels, 14 bit depth) for the duration of development from first cell division until hatching within one of three open-source video microscope systems (OpenVIM [5]). OpenVIMs are robotic digital microscopes for timelapse imaging of developing embryos. Individual embryos were imaged for 30 s at 30 Hz and this acquisition schedule was repeated hourly for the duration of the development of individual embryos. Embryos were maintained in 96 well microtitre plates housed within jacket incubation chambers maintained at 20, 25 or 30 °C located within the OpenVIM. Time lapse video of the development for individual embryos maintained at each temperature, from this study, are accessible at https://doi.org/10.5281/zenodo.1419971.

EmbryoCV, the software component of EmbroPhenomics, produced pixel-wise segmentation of embryos in individual frames at each hourly time point (see Tills et al. (2018) [5] for full image processing pipeline). Movement was calculated from changes in the centre of mass of the embryo on a frame-by-frame basis from its pixel wise segmentation. For each 30 s video, energy proxy traits were measured via signal decomposition of the mean pixel value time-series, within the bounding box of the embryo. Two image resolutions were employed for measuring mean pixel values for different parts of the analysis. Dimensionality reduction was performed using EPTs calculated from mean values of the whole bounding box of the embryo. All other analyses were performed using signal decomposition [26] applied individually to each of 64 mean pixel value signals associated with different regions of the embryo bounding box (8 × 8 grid). The mean of the resultant sixty-four energy spectra was computed and used for subsequent analysis. Heart rate was isolated, quantified and modelled for individual embryos from the signal decomposition step of the EPT calculation (see Tills et al. (2018) [5] for full details of this method). A simplified workflow for calculating energy proxy traits from video is available at—https://doi.org/10.5281/zenodo.4680830 and this can be run online via Google Colab. Realtime video from this study is accessible at—https://doi.org/10.5281/zenodo.4645805.

### Thermodynamic predictions

A Q_10_ = 2 (i.e. a doubling of rate for a 10 °C increase in temperature) [15], was applied to mean phenotype values measured at 20 °C, to make predictions of the responses at 25 and 30 °C. Predicted responses at 25 and 30 °C were made for individual frequencies of EPT, net movement and heart rate. Developmental rates were standardised between temperatures by converting the absolute timing from first cell division, to timing relative to the duration of the period between the first cell division and hatching. The resulting relative timing was used in the calculation of predicted phenotype values at different temperatures across time points to allow direct comparisons between temperatures at equivalent developmental time points.

### Dimensionality reduction

To determine the differences between temperature treatments in the combinatorial signals from EPTs, dimensionality reduction was applied to the mean time-specific data for individual EPTs within each temperature treatment. Temporal frequencies were first binned by a factor of 2 and each resultant bin was normalised to a uniform index of 0—1. PCA was then applied to these data and the resultant eigenvectors to investigate clustering of treatment groups on the basis of linear dimensionality reduction, and also t-stochastic neighbour embedding (t-SNE), a non-linear method. t-SNE was computed on the first 50 principal components (perplexity = 5, theta = 0). The remaining parameters were set to the defaults supplied by Rtsne [27].

### Statistical analysis

All analyses were conducted in R v4.0.3 [28]. PCA was carried out using the package stats [28] and t-SNE was computed using the package Rtsne [27]. Chi squared test was used to test for differences in the number of EPTs, movement and heart rate measures above or below thermodynamic predictions (Q_10_ = 2, Additional file 1 between temperature treatments (Additional file 2, Fig. 1a). Proportional difference in overall levels of EPT, movement and heart rate from thermodynamic predictions (Additional file 3, Fig. 1b) were tested using an ANOVA. Test of the relative sensitivity of different traits (EPTs at different frequencies, movement and heart rate) across developmental periods (Additional file 4, Fig. 2) were tested using an ANOVA. Multivariate Analysis of Variance (MANOVA) was used to test for differences in the first 50 principal components calculated from a PCA using mean treatment hourly time point EPT data reduced from 300 frequencies to 150 bins (Additional file 5, Fig. 3a). Subsequent t-SNE dimensionality reduction was performed on the first 50 PCs from this PCA (Additional file 6, Fig. 3b).

### Data and material availability

Downstream experimental data are provided as supplementary material. Time-lapse video from the study is available at: https://doi.org/10.5281/zenodo.1419971. Realtime study video from different treatments are available at: https://doi.org/10.5281/zenodo.4645805. Worked examples of energy proxy trait calculation compatible with Google Colab are available at: https://doi.org/10.5281/zenodo.4680830.

## Supplementary information

**Additional file 1** Counts of number of traits within the categories energy, heart rate and movement at 25 and 30 °C with values either above or below the values predicted from Q10=2 applied to measurements at 20 °C

**Additional file 2** Predicted (Q10=2) and observed trait values for movement, heart rate and energy during different periods of development in 25 and 30 °C.

**Additional file 3** Overall levels of deviation in heart rate, energy and movement from Q10=2 predictions.

**Additional file 4** Individual deviation of heart rate, energy and movement traits from Q10=2 predictions during different periods of development at 25 and 30 °C.

**Additional file 5** Principal component sample weightings from a PCA of mean EPT spectra for Radix balthica embryos cultured at 20, 25 and 30 °C at hourly time points.

**Additional file 6** Sample loadings for a T-distributed stochastic neighbour embedding (t-SNE) analysis performed on loadings from PCs1-50 from a PCA performed on hourly EPT spectra for Radix balthica cultured at 20, 25 and 30 °C.

**Additional file 7** Growth rate and total energy proxy trait levels for embryos maintained in different treatment temperatures.

**Additional file 8** Time series of total EPT energy for an individual embryo cultured at 20 °C for the duration of its embryonic development. Annotations for manually ascertained developmental events are added A) onset of ciliary driven spinning, B) onset of muscular crawling and C) attachment of foot to the egg capsule.

## Data Availability

Data are made available as Supplementary Material and on the EmbryoPhenomics Zenodo data community: https://zenodo.org/communities/embryophenomics
