# Supplementary figures and images for: Spectral phenotyping of embryonic development reveals integrative thermodynamic responses
Source: BMC Bioinformatics. 2021 May 6;22:232. doi: 10.1186/s12859-021-04152-1 (PMC8101172; doi:10.1186/s12859-021-04152-1)

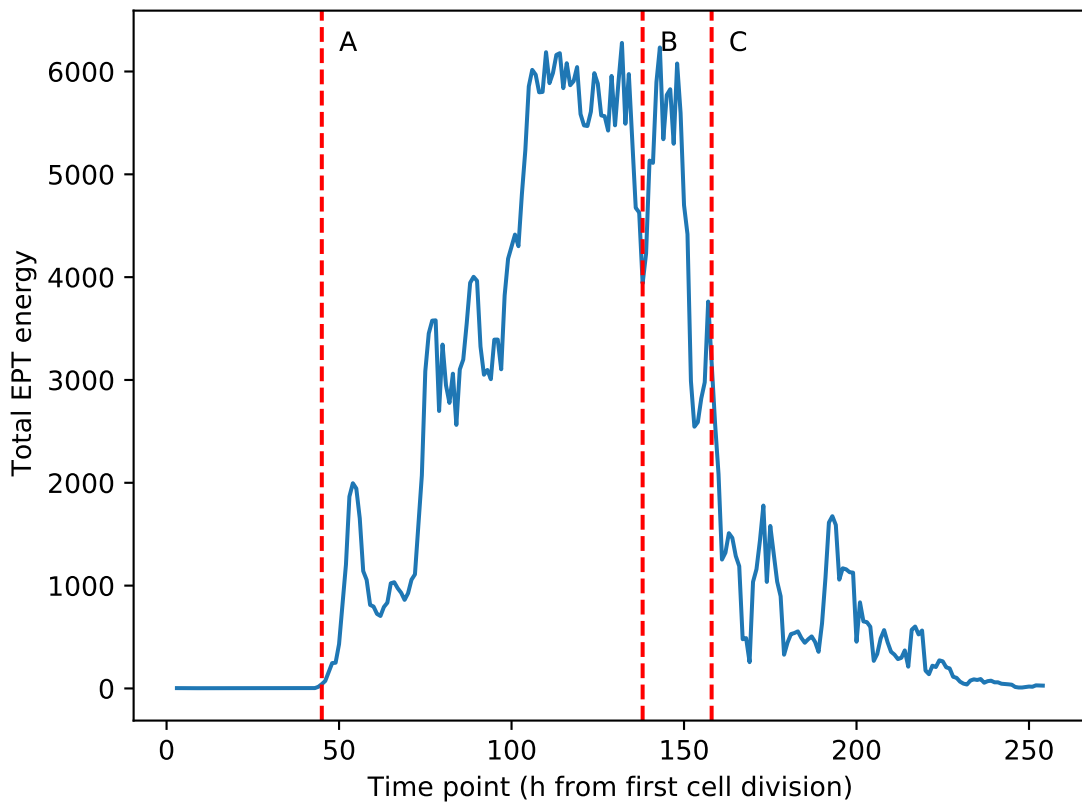

Supplement: Supplementary file 8 — Additional file 8 Time series of total EPT energy for an individual embryo cultured at 20 °C for the duration of its embryonic development. Annotations for manually ascertained developmental events are added A) onset of ciliary driven spinning, B) onset of muscular crawling and C) attachment of foot to the egg capsule. [file 12859_2021_4152_MOESM8_ESM.pdf]
